# Modifications of the Dental Hard Tissues in the Cervical Area of Occlusally Overloaded Teeth Identified Using Optical Coherence Tomography

**DOI:** 10.3390/medicina58060702

**Published:** 2022-05-25

**Authors:** Andreea Stănuși, Mihaela Ionescu, Cristina Cerbulescu, Sanda Mihaela Popescu, Eugen Osiac, Răzvan Mercuț, Monica Scrieciu, Roxana Maria Pascu, Adrian Ştefan Stănuși, Veronica Mercuț

**Affiliations:** 1 Department of Prosthetic Dentistry, University of Medicine and Pharmacy of Craiova, 200349 Craiova, Romania; andreeacazan22@gmail.com (A.S.); pascuroxana81@yahoo.com (R.M.P.); adrian.stefan.stanusi@gmail.com (A.Ş.S.); veronica.mercut@umfcv.ro (V.M.); 2 Department of Medical Informatics and Biostatistics, University of Medicine and Pharmacy of Craiova, 200349 Craiova, Romania; cris_gabriela@yahoo.com; 3 Department of Oral Rehabilitation, University of Medicine and Pharmacy of Craiova, 200349 Craiova, Romania; sanda.popescu@umfcv.ro; 4 Department of Biophysics, University of Medicine and Pharmacy of Craiova, 200349 Craiova, Romania; eugen.osiac@umfcv.ro; 5 Department of Plastic Surgery and Reconstructive Microsurgery, University of Medicine and Pharmacy of Craiova, 200349 Craiova, Romania; razvan.mercut@umfcv.ro

**Keywords:** optical coherence tomography, OCT, non-carious cervical lesion, tooth cracks, occlusal overload

## Abstract

*Background and objectives*: Occlusal overloads produce a series of manifestations in teeth, especially attrition and non-carious cervical lesions (NCCL). Optical Coherence Tomography (OCT) can highlight and evaluate tooth lesions. The aim of this study was to examine the changes of dental hard tissues in the cervical area because of occlusal overload by macroscopic examination and using in vitro Swept Source OCT examination. *Materials and Methods*: The study was performed on 21 extracted teeth with occlusal trauma. After macroscopic and OCT examination, the 2D OCT images were transformed into 3D images using ImageJ software. Statistical analysis of macroscopic and OCT images was performed with Statistical Package for Social Sciences. *Results*: On 21 teeth, 88 cervical lesions (cracks) were identified. Upper premolars with an occlusal Smith and Knight tooth wear score of 2 had the most NCCL. Statistical analysis revealed significant differences in the median widths of cervical lesions between teeth with score 1 and score 3. Additionally, we obtained statistically significant differences in median widths between the buccal and oral surfaces. *Conclusions*: These cracks can be considered precursors of NCCL. NCCL can be located on dental surfaces in the cervical area other than the buccal one. A 3D reconstruction of OCT images emphasized that cracks are located especially at enamel level, evolving towards the enamel–dentin junction, with multiple ramifications.

## 1. Introduction

Occlusal overloads produce a series of manifestations in teeth, of which a special place is occupied by attrition and non-carious cervical lesions (NCCL). In terms of attrition, the mechanism of hard tissue loss seems easy to understand [1], whereas the mechanism of production of NCCL is not very clear.

In 1991, Grippo used the term “abfraction” to define NCCL and considered them a consequence of excessive occlusal forces [2]. He described five types of NCCL in enamel (hairline cracks, striations, saucer-shaped lesions, semilunar-shaped lesions, cusp-tip invagination) and ten types in dentin (gingival, circumferential, multiple, sub-gingival, lingual, interproximal, alternate, angular, crown margin, restoration margin) [2].

In 2019, a group of 15 experts from the European Dental Caries Research Organization and the Cariology Research Group of the International Association for Research in Dental Medicine (IADR) met at a workshop in Frankfurt to define the terminology associated with erosive dental wear and dental caries. They unanimously agreed that the term “abfraction” is not recommended to be used because there is not enough information to show that this form of dental wear is caused only by excessive occlusal forces [3]. Currently, the terminology used to name these lesions is still a subject of debate between groups of specialists [3,4]. In 2022, Nascimento et al. concluded that occlusal factors may be involved in NCCL generation [5].

Optical Coherence Tomography (OCT) is an advanced biomedical imaging technique that allows visualization of the internal biological structure in a non-invasive manner and has multiple applications in numerous medicine fields [6]. The first in vitro and in vivo OCT images of hard and soft dental tissues were obtained by Colston in 1998 [7]. The micrometric resolution of this technology and the depth of penetration measured in millimeters determined the use of OCT devices for dental research in caries [8], demineralization [9], cracks [1,10,11], fractures, tooth defects [12] and tooth wear [1].

Currently a higher number of publications describe the applications of OCT and the advantages of this method in highlighting and evaluating tooth lesions [1,13]. Among these tooth lesions, tooth wear and especially NCCL arouse the greatest interest from specialists due to incompletely elucidated ethiopathogenic mechanisms.

The aim of this study was to highlight the changes of dental hard tissues in the cervical area as a consequence of occlusal teeth overload, using in vitro Swept Source Optical Coherence Tomography (SS-OCT), to determine which teeth are most affected, the association of cervical lesions with the degree of occlusal wear, and to identify the most affected axial surfaces. Furthermore, the study aimed to assess more accurately the lesions in the hard tissues by tridimensional reconstruction of images using the ImageJ program.

## 2. Materials and Methods

### 2.1. Teeth Prelevation and Preparation

The study was performed on 21 teeth without endodontic treatments, fillings or carious lesions in the cervical areas, represented by 7 upper molars, 8 lower molars, 4 upper premolars and 2 lower premolars. The study was approved by the Ethics Committee of University of Medicine and Pharmacy from Craiova, Romania (no. 20/12 February 2021), and it is in accordance with relevant guidelines and regulations. Patients provided written informed consent for dental treatment and participation in the study.

The teeth included in the study were extracted for various reasons from 13 patients over the age of 60, who presented for diagnostic and treatment in Dental Prosthetics Clinic of the Faculty of Dentistry, University of Medicine and Pharmacy of Craiova, during March–April 2021.

The inclusion criteria in the study for the patients were: (1) informed consent to participate in the study, (2) diagnosis of extended partial edentulism, with multiple occlusal imbalances (unevenness of the occlusion plane, premature contacts in static cranio-mandibular positions and occlusal interferences in mandibular dynamics) established following clinical examination, examination of casts and radiological examination (orthopantomogram), and (3) teeth to be extracted showed low support capacity or various odonto-periodontal diseases in which conservative treatments could not be performed and could not be taken into account for a prosthetic rehabilitation.

Of these, teeth that showed conclusive signs of occlusal trauma (advanced alveolar atrophy after which the crown/root ratio was greater than 1, mesio-distal inclination of teeth more than 45 degrees and tooth mobility) were selected for this study.

All teeth selected for this study had antagonist teeth, while they were in function in the oral cavity. Thereby, these teeth could be considered to have been subjected to occlusal overloads because the masticatory forces were supported by a smaller number of teeth in the functional processes of mastication and swallowing.

The examination of patients and teeth extractions were performed by 6 dentists specialized in general dentistry and dental prosthetics, authors of the study from the Department of Prosthetic Dentistry and Oral Rehabilitation, according to conventional techniques, as little traumatic as possible, to avoid formation of additional cracks [14].

The extracted teeth were immersed in hydrogen peroxide solution 10% for 10 min to remove organic residues. Subsequently, the teeth were scaled and brushed and kept in saline solution NaCl 0.9% until examination, to prevent dehydration and formation of additional cracks.

Selected teeth were examined macroscopically, and occlusal tooth wear was evaluated according to the Smith and Knight tooth wear score [15]. The presence of NCCL was also registered.

Tooth samples were prepared for SS-OCT examination according to a protocol used in other studies [1]. The teeth were fixed in high-grade silicone (Zetaplus L Intro KIt, Zhermack, Badia Polesine, Italy) for OCT examination, leaving the surface to be examined exposed. Teeth were positioned so the light beam would fall perpendicularly on the examined surfaces.

### 2.2. Macroscopic and 2D OCT Examination of Teeth

The macroscopic examination of teeth was performed using photographs taken with Canon DSLR 600EOS (Tokyo, Japan).

The SS-OCT examination was performed using a system developed by Thorlabs (OCS1300SS, Thorlabs) with a laser beam characterized by a central wavelength of 1310 nm, a spectral band of 100 nm and an average power of 12 mW. The system was used for 2D in vitro scanning of dental surfaces, and its resolution was 12 µm for the axial resolution and 15 µm for the lateral one. The system allowed the examination of samples measuring 6 mm × 5 mm × 1.5 mm (length, width and depth) in about 30 s using the charge-coupled device type detector [16].

The scan was performed in the cervical area on all axial faces of teeth, obtaining approximately 500 2D images for each scanned surface. For the quantitative evaluation, the refractive index of 1.58 was used, representing an average between the refractive index of enamel (1.63) and dentin (1.54) [17]. Representative images were chosen to evaluate the cracks. We measured the width of cervical lesions in mm, using the Thorlabs program. Three dentists and an expert in OCT images have independently measured each crack. One dentist and the OCT expert repeated the measurements, with a two-week interval between measurements, and teeth re-arranged in a randomized order. Their results were used to compute two intra-reliability scores, using the Intraclass Correlation Coefficient (ICC). Inter-rater reliability was computed also using the ICC. Final values were obtained as the average values for all 4 raters.

Cracks in the dental hard tissues and areas of structural alteration appeared as signals of high intensity, differentiated by the enamel and dentin mass. The high intensity signal from the cracks was determined by a higher refraction due to the interruption of the tissue continuity. The high intensity signal from structural alteration areas was determined by multiple reflections as a result of the signal destructuring. In these areas, tissue damage was caused by demineralization and stress concentration.

### 2.3. Statistical Analysis of Macroscopic and OCT Images

Tooth data were first grouped using Microsoft Excel. The same program was used for the basic descriptive analysis. For group comparisons, continuous variables were expressed as mean ± STDEV (standard deviation). We used Statistical Package for Social Sciences (SPSS), version 20 (IBM Corp.) to apply statistical tests upon our parameters. Correlations were analyzed based on Chi-square test. Mann–Whitney U and Kruskal–Wallis H tests were used for group distributions, based on the results of the Shapiro–Wilk normality test. Post hoc analysis was based on Dunn’s (1964) procedure with a Bonferroni correction for multiple comparisons, and the value *p* < 0.05 represented the statistically significance threshold.

### 2.4. Transforming 2D OCT Images into 3D Images Using ImageJ Software

Three-dimensional reconstruction ensures the assessment of the disposition of the cracks in the hard dental tissues. Three-dimensional images provide information on changes in hard dental structures from multiple angles along the 3 axes [18].

The 2D OCT images (JPEG image format) were further transformed into 3D images and 3D volume viewer images using ImageJ software.

ImageJ was developed on Mac OSX using its built-in editor and Java compiler.

The source code is freely available (tutorial). The author, Wayne Rasband, is a Special Volunteer at the National Institute of Mental Health, Bethesda, MD, USA (1997). This program does geometric transformations such as scaling, rotation and flips [19].

## 3. Results

### 3.1. Results of Macroscopic Images Analysis

The 21 teeth included in the study were represented by 10 teeth from the mandibular arches and 11 teeth from the maxillary arches. In other words, the study was performed on 15 molars (7 upper molars, 8 lower molars) and 6 premolars (2 lower premolars, 4 premolars).

Following direct examination and macroscopic images analysis of the 21 teeth, the diagnosis of occlusal wear of various degrees was established for all teeth as well as presence of NCCL (Table 1).

According to Table 1, upper premolars with an occlusal Smith and Knight tooth wear score of 2 had the most NCCL.

From all the images obtained for this study, macroscopic images of an upper first premolar (PM1), an upper second premolar (PM2) and a lower second molar (m2) will be presented.

**Figure 1:**
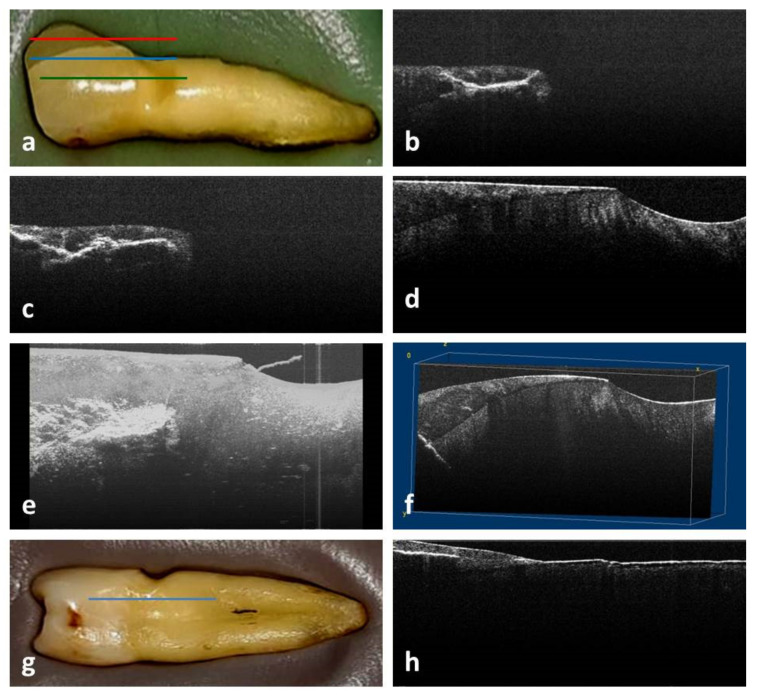
PM1 examination: (**a**) macroscopic aspect of buccal surface; (**b**) 2D OCT image of buccal area with concave fissure; (**c**) 2D OCT image of buccal area with crack in dentin; (**d**) 2D OCT image of buccal area with cracks perpendicular to the bottom of NCCL; (**e**) 3D image of buccal surface in the cervical area; (**f**) 3D volume viewer image of buccal surface in the cervical area; (**g**) macroscopic aspect of mesial surface; (**h**) 2D OCT image of mesial area with crack in enamel.

In the area of the buccal cusp of PM1, the occlusal Smith and Knight tooth wear score had a value of 2. In the buccal cervical area, an NCCL of medium depth, wedge-shaped, at the enamel–cement junction, was identified by visual examination (Figure 1a).

**Figure 2:**
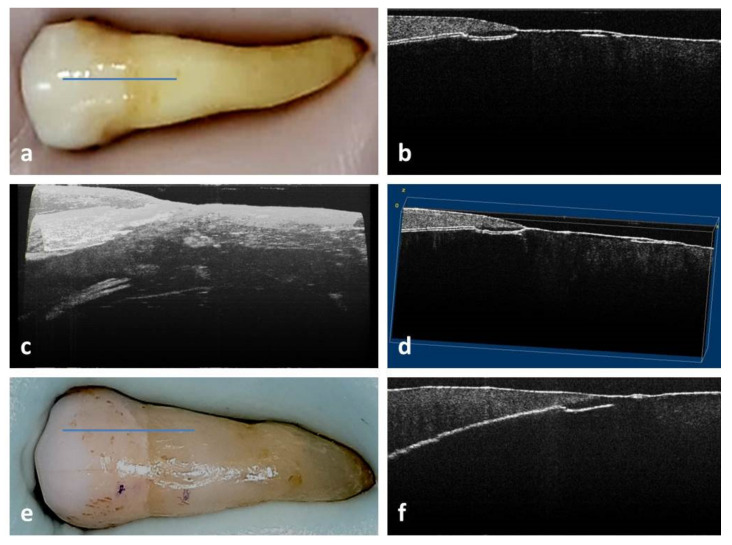
PM2 examination: (**a**) macroscopic aspect of buccal surface; (**b**) 2D OCT image of buccal area with crack at enamel–dentin junction; (**c**) 3D image of buccal surface in the cervical area; (**d**) 3D volume viewer image of buccal surface in the cervical area; (**e**) macroscopic aspect of oral surface; (**f**) 2D OCT image of oral area with crack at the enamel–dentin junction.

In the case of PM2, the occlusal Smith and Knight tooth wear score had a value of 2, and no NCCL was identified by visual examination (Figure 2a).

**Figure 3:**
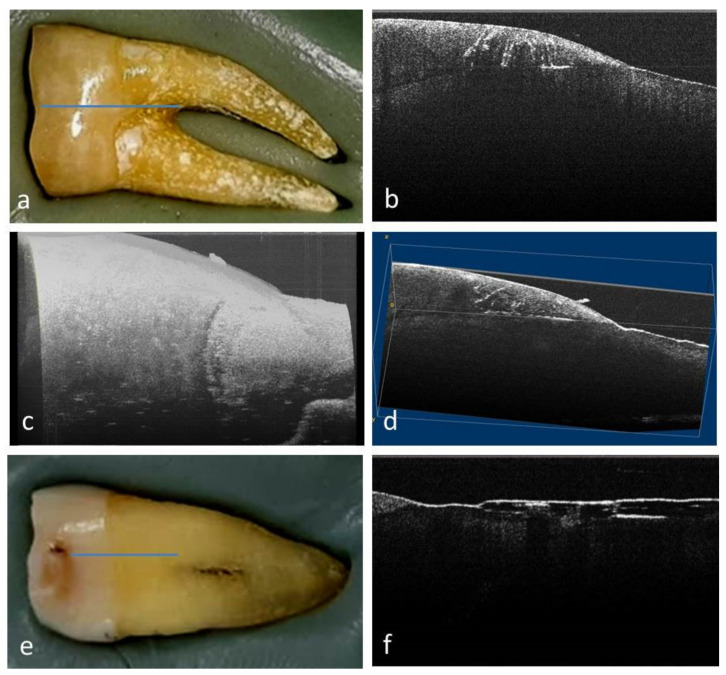
m2 examination: (**a**) macroscopic aspect of buccal surface; (**b**) 2D OCT image of buccal area with crack at enamel–dentin junction and cracks of radial appearance in the enamel; (**c**) 3D image of buccal surface in the cervical area; (**d**) 3D volume viewer image of buccal surface in the cervical area; (**e**) macroscopic aspect of mesial surface; (**f**) 2D OCT image of mesial area with crack in cement.

Tooth m2 presented an occlusal Smith and Knight tooth wear score of 3, and no NCCL was diagnosed by visual examination (Figure 3a).

### 3.2. Results of 2D OCT Images Interpretation

Following SS-OCT examination, besides the 4 NCCL described in the macroscopic examination, another NCCL on the oral face of an upper premolar and 83 cracks with various locations and widths were identified, resulting in a total of 88 cervical lesions (Table 2). Of these, 46 were recorded on the maxillary teeth and 42 on the mandibular teeth, of which there were 68 on the molars and 20 on the premolars.

The distribution of cervical lesions according to the Smith and Knight tooth wear score was as follows: 48 cervical lesions were recorded on teeth with an occlusal Smith and Knight tooth wear score of 1 with an average of 4.36 lesions per tooth, 15 cervical lesions on teeth with an occlusal Smith and Knight tooth wear score of 2 with an average of 3.71 lesions per tooth and 25 cervical lesions on teeth with an occlusal Smith and Knight tooth wear score of 3 with an average of 4.16 lesions per tooth (Table 2).

The most affected dental surfaces were the buccal ones, with 40 cervical lesions, followed by the mesial surfaces with 19 cervical lesions, the oral faces with 15 cervical lesions and the distal ones with 14 cervical lesions. Each surface presented either unique lesions or multiple lesions. A lesion was considered unique when it was the only lesion present on one side; a lesion was considered as part of a set of multiple lesions when there were at least two lesions present on the same side. Our study lot comprised 42 multiple lesions (representing 47.73%) and 46 unique lesions (52.27%).

Depending on the degree of damage to the hard dental tissues; 76 cervical lesions were in the enamel, 7 cervical lesions were located at the enamel–dentin junction and 5 were located in the cement

Regarding the width of cracks, the situation is presented in Table 3.

From all the 2D OCT images obtained for this study, those of PM1, PM2 and m2 will be presented.

In the case of PM1, the 2D OCT images of buccal cervical area highlighted the existence of a concave fissure, located coronary from the NCCL, in the enamel, which continues at the enamel–dentin junction, corresponding to the red (Figure 1b) and blue (Figure 1c) lines in macroscopic image (Figure 1a). Additionally, there was a crack in the depth of the coronary dentin (Figure 1c). In addition, it was observed that there were several cracks perpendicular to the bottom of the cavity of the NCCL and to its edges, which propagate in the depth of the hard tissues (Figure 1d). Cracks were also observed on the mesial and distal (Figure 1g,h) surfaces of this tooth. These were located mainly in the enamel with evolution towards the dentin.

In the case of PM2, the 2D OCT images of the buccal cervical area highlighted the existence of a straight crack at the enamel–dentin junction (Figure 2b). Initially, at this crack the OCT signal was well outlined, but towards the middle of the buccal surface a doubling of the signal was noticed, interpreted as an incomplete detachment of the enamel fragment. Examination of the mesial half of the buccal surface showed that the crack had become concave and was positioned below the enamel–dentin junction. Unlike the tooth described above, it showed a crack on the oral surface at the junction of enamel–dentin (Figure 2e,f).

For tooth m2, the 2D OCT examination of the buccal cervical area highlighted the existence of several cracks, positioned at the enamel–dentin junction and others with a radial appearance, located in the enamel (Figure 3b). On this tooth, a crack on the mesial face was also highlighted (Figure 3e,f) in the cement.

### 3.3. Statistical Results

The statistical results are based on the macroscopic examination of the 2D OCT images.

Intra-rater reliability involved the analysis of two persons’ measurements of all cracks, following a second measurement process after a 2-week interval. ICC analysis showed a very good intra-rater agreement (0.990; 95% CI: 0.985–0.993 for the OCT expert, and 0.985; 95% CI: 0.977–0.990 for the dentist). Inter-class correlation coefficient analysis indicated also a very good inter-rater agreement (0.981; 95% CI: 0.973–0.986) when comparing the measurements of all 4 persons involved in this phase of the study. The final values represented the average measurements of all 4 raters.

A Chi-square test was conducted between the presence of multiple lesions and tooth surfaces. All expected cell frequencies were greater than five. There was a moderately strong association between these variables (Cramer’s V = 0.313), which was statistically significant (*p* = 0.035).

Similar tests were conducted to identify potential correlations between the presence of multiple cracks and tooth type (molar, premolar), tooth position (maxillary and mandible) and the degree of tooth wear (expressed by the occlusal Smith and Knight tooth wear score), but no statistically significant associations were obtained (*p* > 0.05).

Mann–Whitney U tests were run to determine if there were differences in cracks’ size between premolars and molars, lower and upper teeth, as well as teeth with and without NCCL. Distributions of the cracks’ width for these groups were similar, as assessed by visual inspection. Differences were statistically significant only between maxillary and mandibular teeth, where we identified that the width cracks on lower teeth varied from 0.027 mm to 0.489 mm, with a mean of 0.184 ± 0.174, and the width cracks on upper teeth varied from 0.022 mm to 0.421 mm, with a mean of 0.094 ± 0.126. Results are indicated in Table 4.

The distribution of crack width according to the degree of occlusal tooth wear showed that the teeth with an occlusal Smith and Knight tooth wear score of 2 presented the largest cervical lesions, with a mean of 0.176 ± 0.162. Those were followed by teeth with an occlusal Smith and Knight tooth wear score of 3, with a mean of 0.168 ± 0.192, and teeth with an occlusal Smith and Knight tooth wear score of 1, with a mean of 0.108 ± 0.129. For teeth with an occlusal Smith and Knight tooth wear score of 1, cervical lesions seem to be more uniform in width, while for teeth with scores 2 and 3, cervical lesions present various widths. For groups of teeth defined by the occlusal wear score, we obtained statistically significant differences in cracks’ size (Table 4). Thus, we conducted pairwise comparisons based on Dunn’s (1964) procedure with a Bonferroni correction for multiple comparisons. Statistical significance was accepted at *p* < 0.0166 level for the three levels of tooth wear defined by the Smith and Knight score. This post hoc analysis revealed statistically significant differences in median widths between the groups with index 1 and index 3 (*p* = 0.0160), but not between any other group combination.

Regarding crack location on dental surfaces, buccal dental surfaces are more likely to have multiple cervical lesions (22), followed by mesial surfaces (10), distal (6) and oral surfaces (4). There are statistically significant differences in cracks’ width between these groups of teeth (Table 4). Thus, we conducted pairwise comparisons based on Dunn’s (1964) procedure with a Bonferroni correction for multiple comparisons. Statistical significance was accepted at *p* < 0.0083 level (for the four possible cracks’ location). This post hoc analysis revealed significant differences in median widths between the buccal and oral sides (*p* = 0.007), but not between any other group combination.

There was no statistically significant difference regarding the width of cracks from different locations (enamel, enamel–dentin junction, cement), *p* > 0.05.

### 3.4. Results of 3D Reconstruction Images

The 3D reconstruction made using ImageJ program, on the 3 axes of the 2D OCT images, confirmed the presence of the cervical lesions and revealed their location and orientation in space.

In the case of PM1, the 3D reconstruction revealed a buccal cervical lesion with a “wedge” appearance, from the level of which towards the occlusal was observed the propagation of a crack in the thickness of the hard tissues, with multiple ramifications. (Figure 1e,f)

In the case of PM2, the 3D reconstruction revealed a surface crack, in the thickness of the hard tissues, with a semicircular appearance, going from the enamel–cement junction to the occlusal and from surface into depth (Figure 2c,d).

In the case of m2, the 3D reconstruction revealed multiple radial cracks, oriented from surface into depth, stopping at the level of the enamel–dentin junction. At the enamel–dentin junction, another crack was noticed, parallel to the surface of the enamel. The 3D image brings together all the aspects recorded in the 2D OCT examination (Figure 3c,d).

## 4. Discussion

This study aimed to highlight the effects of occlusal overloads on hard tissues in the cervical area of teeth with varying degrees of occlusal wear. For this, a macroscopic examination and 2D OCT examination were performed. The additional objective of this study was to process OCT images from 2D to 3D with the ImageJ program, for a more accurate assessment of the distribution and orientation of the detected cervical lesions. Volumetric reconstructions and 2D image processing techniques are commonly employed in various medical fields in order to increase the accuracy of content analysis or object detection [20,21,22].

The effects of occlusal overloads highlighted in the form of dental wear (attrition and NCCL) [23,24], in the form of dental fissures and in the form of disorders of trophicity of the dental pulp [25,26] are also discussed in other publications [27].

The present study was performed on 21 extracted teeth with occlusal wear of various degrees. The quantification of occlusal wear was performed using the Smith and Knight tooth wear score, a classic index used in many research studies to assess the degree of tooth wear regardless of the etiology involved. NCCL were identified through macroscopic and OCT examination and cracks were identified using only OCT examination. Along with NCCL as a direct consequence of occlusal overloads in the dental hard tissues, a series of cracks in the form of bright areas were highlighted, on a gray background. The diagnosis of dental cracks by current methods is difficult to achieve due to their location. Shimada showed that SS-OCT was an effective diagnostic method for dental cracks [7] and presented in a study conducted in 2020 the OCT image of an upper incisor with a crack on the buccal surface. The crack appeared as a white line that affected the entire thickness of the enamel. Numerous enamel cracks of varying widths, identified as white lines, were observed in the present study. These white lines were caused by the penetration of the OCT signal into the deposits inside them, formed by protein-rich fluids and involved in a self-healing mechanism [28].

Although the study initially aimed to highlight NCCL in teeth subjected to occlusal overloads, the large number of cracks and their morphology foreshadowing future NCCL, led to their inclusion in the effects of occlusal overloads and referred to them during the study as of “cervical lesions”. In fact, Grippo, in 1991, described NCCL as “hairline lesions” that can be located on all sides of the teeth [2]. Prior to Grippo, the presence of hard tissue cracks was included in 1964 by Cameron [29] in cracked tooth syndrome. The author associated cracked tooth syndrome with wear on the occlusal surfaces, considered as evidence of interference, and with interceptive occlusive contacts from bruxism. At the same time, he associated cracked tooth syndrome with high cusps and deep grooves, when excessive muscle strength is present. In 2020, Roma [30] associated dental cracks with occlusal overloads and restorative treatments.

There are other studies in literature that focused on the association between occlusal wear and the presence of lesions in the cervical area of the respective teeth. Most studies are performed by Finite Element Analysis (FEA) and show that the less the occlusal morphology is affected by wear, the higher the stress concentration in the cervical areas of the teeth, especially on the buccal surfaces [23,24].

The present study revealed the highest number of cervical lesions to be in teeth with an occlusal Smith and Knight tooth wear score of 1. The explanation could be given by the presence of premature contacts and occlusal interferences specific to teeth with primary or slightly modified occlusal morphology. The same explanation is given by Troia in 2021 [31] and Chandrathara in 2020 [32].

In contrast, the study showed a slight increase in the number of cervical lesions in teeth with an occlusal Smith and Knight tooth wear score of 3 compared to teeth with an occlusal Smith and Knight tooth wear score of 2. A possible explanation would be that score 2 occlusal wear has an adaptive character, whereas in score 3 occlusal wear, clenching and grinding can be invoked in addition to excessive occlusal forces [33,34]. The same association between reduced occlusal wear and the presence of lesions in the cervical area in the form of NCCL was reported by Duangthip in 2017 [35], Lyons in 2001 [36] and Telles in 2000 [37]. On the other hand, Estafan [38] showed that there was no correlation between NCCL and occlusal wear.

Although this is not a study focused on establishing the prevalence of cervical lesions, it showed that the most affected teeth were premolars. The result overlaps with studies on the prevalence of NCCL that have shown a preferential impairment of premolars [39,40,41].

Regarding the degree of damage to the axial faces, most manifestations of occlusal stress were identified in the buccal cervical area, followed by the mesial, oral and distal cervical area, respectively. This result is consistent with the preferential location of NCCL on the buccal surfaces of the teeth. Classical forms of abfraction lesions have been present mainly on the buccal aspects of teeth, although manifestations of occlusal overloads have been located in other cervical areas, which shows that there are several factors involved. The distribution of cervical lesions suggests the presence of morphological features of the enamel in these areas and the fact that several factors are involved in the genesis of NCCL.

Regarding the width of these cervical lesions, mandibular teeth showed the highest variety of damage. Hughes analyzed the cracks in the cervical enamel and demonstrated in a 2009 study [42] that the direction of propagation of cracks during the functionality of the teeth on the dental arch was from the outside to the inside of the hard tissues. In the present study, most of the cracks identified in the cervical enamel of the examined teeth did not exceed the enamel–dentin junction. Imbeni conducted a study in 2005 in which he demonstrated that for cracks initiated in enamel, the evolution was stopped when the enamel–dentin junction was reached, or a minimum penetration (0.01 mm) was achieved in the dentinal mantle [40]. Stopping the evolution of cracks from enamel could be due to the biomechanical properties of the enamel–dentin junction. The hardness of this junction is 5–10 times higher than that of enamel, but 75% lower than that of dentin [39,40,41]. The enamel–dentin junction can dampen a large amount of stress before allowing the spread of cracks in the dentin or can even completely stop the evolution of a crack in the dentin [42,43,44,45].

In the last part of the study presented, the 2D OCT images were transformed into 3D images and 3D volume viewer images, using ImageJ software. This 3D format allows a visualization of the lesions in all dental structures. To our knowledge, there are no studies in which the reconstruction of 3D images and 3D volume viewer of 2D OCT images was performed.

The limitations of the study are represented by the small number of teeth examined.

## 5. Conclusions

The study allowed the identification by macroscopic and OCT examination of the effects of occlusal overloads in the cervical areas of teeth in the form of NCCL and cracks located mostly on the vestibular surfaces of teeth.

The present study performed a complete assessment of the lesions in the cervical area of teeth subjected to occlusal overloads and found that occlusal factors may be associated with NCCL. The similarity of some of the dental cracks to that of the NCCL has been highlighted, so that these cracks can be considered precursors of NCCL. The study confirmed the possibility of NCCL on dental surfaces in the cervical area other than the buccal one.

A 3D reconstruction of OCT images performed using the ImageJ software application showed that cracks are present on all axial sides, especially in dental enamel, with an evolution towards the enamel–dentin junction, and with multiple ramifications.

## Figures and Tables

**Table 1:** NCCL distribution according to tooth type and occlusal wear index.

	Lower Molars	Upper Molars	Lower Premolars	Upper Premolars	Total No of Teeth
Nr. of teeth included in study	8	7	2	4	21
Occlusal Smith and Knight tooth wear score 1	3	6	0	2	11
Occlusal Smith and Knight tooth wear score 2	2	0	0	2	4
Occlusal Smith and Knight tooth wear score 3	3	1	2	0	6
NCCL	0	1	1	2	4

**Table 2:** Cervical lesions distribution according to occlusal wear score and affected surface (B = buccal, O = oral, M = mesial, D = distal).

Occlusal Smith and Knight Tooth Wear Score	No of Teeth (%)	No of Cervical Lesions (%)				Total No of Cervical Lesions (%)
		B	O	M	D	
Score 1	11 (52.38)	21 (52.50)	10 (66.67)	10 (52.63)	7 (50.00)	48 (54.55)
Score 2	4 (19.05)	6 (15.00)	3 (20.00)	4 (21.05)	2 (14.29)	15 (17.05)
Score 3	6 (28.57)	13 (32.50)	2 (13.33)	5 (26.32)	5 (35.71)	25 (28.41)
Total	21 (100)	40 (100)	15 (100)	19 (100)	14 (100)	88 (100)

**Table 3:** Width of cracks on dental surfaces (mm), (B = buccal, O = oral, M = mesial, D = distal).

	Premolar			Molar		
	Mean ± SD	Maximum	Minimum	Mean ± SD	Maximum	Minimum
B	0.050 ± 0.017	0.068	0.031	0.111 ± 0.151	0.489	0.020
O	0.254 ± 0.226	0.457	0.056	0.232 ± 0.158	0.421	0.035
M	0.160 ± 0.205	0.468	0.036	0.117 ± 0.136	0.390	0.020
D	0.050 ± 0.012	0.059	0.032	0.191 ± 0.193	0.487	0.031

**Table 4:** Statistical results regarding the analysis of cracks’ width according to various groups of teeth (* Mann–Whitney U test; ** Kruskal–Wallis H test).

	Tooth Type	Tooth Position	NCCL	Tooth Wear	Crack Type	Crack Location
Crack width	U = 581 *p* * = 0.324	U = 412 *p* * < 0.0005	U = 211.5 *p* * = 0.398	χ^2^(3) = 10.205 *p* ** = 0.006	χ^2^(3) = 1.757 *p* ** = 0.415	χ^2^(4) = 11.008 *p* ** = 0.012

## Data Availability

The datasets used and/or analyzed during the current study are available from the corresponding author on reasonable request.
